# Linking the gut microbiome to microglial activation in opioid use disorder

**DOI:** 10.3389/fnins.2022.1050661

**Published:** 2022-12-16

**Authors:** Danielle Antoine, Greeshma Venigalla, Bridget Truitt, Sabita Roy

**Affiliations:** ^1^Department of Surgery, Miller School of Medicine, University of Miami, Miami, FL, United States; ^2^Department of Neuroscience, Miller School of Medicine, University of Miami, Miami, FL, United States

**Keywords:** microglia, microbiome, SUD, opioids, OUD

## Abstract

Substance use disorder (SUD) is a physical and psychological disorder globally prevalent today that has resulted in over 107,000 drug overdose deaths in 2021 in the United States alone. This manuscript reviews the potential relationship between opioid use disorder (OUD), a prevalent subset of SUD, and the microglia, the resident macrophages of the central nervous system (CNS), as they have been found to become significantly more activated during opioid exposure. The inflammatory response mediated by the microglia could contribute to the pathophysiology of SUDs, in particular OUD. Further understanding of the microglia and how they respond to not only signals in the CNS but also signals from other areas of the body, such as the gut microbiome, could explain how the microglia are involved in drug use. Several studies have shown extensive communication between the gut microbiome and the microglia, which may be an important factor in the initiation and development of OUD. Particularly, strategies seeking to manipulate and restore the gut microbiome have been shown to reduce microglial activation and attenuate inflammation. In this review, we discuss the evidence for a link between the microglia and OUD and how the gut microbiome might influence microglial activation to drive the disorder and its associated behaviors. Understanding this connection between microglia and the gut microbiome in the context of drug use may present additional therapeutic targets to treat the different stages of drug use.

## 1 Introduction

The complex relationship between drugs of abuse, microglia, and the gut microbiome is important to investigate due to the nature of substance use disorder (SUD) as a pressing psychiatric disorder globally prevalent today. In 2021, there were more than 107,000 drug overdose deaths in the U.S., including those caused by both illicit drugs and prescription opioids (Ahmad and Rossen, 2020). SUD is a chronic relapsing disease characterized by compulsive drug taking behavior despite the negative social, occupational, and health-related consequences. Commonly abused substances include methamphetamine, cocaine, and opioids (Ahmad and Rossen, 2020). While all drugs of abuse can be harmful and cause the onset of SUD, of particular importance in the United States are opioids. The over-prescription of opioids for pain management has led to an opioid epidemic with millions of people suffering from opioid use disorder (OUD), a subset of SUD, in which the repeated use and need of opioids cause failure to meet the demands and responsibilities of everyday life. OUD in the United States alone has caused over 71,000 overdose fatalities in 2021 alone, making up approximately 75% of all drug overdose deaths that year (Centers for Disease Control, [CDC], 2021). As of 2022, approximately 3 million individuals currently have or previously have had OUD (Azadfard et al., 2022), largely due to the widespread over-prescription of opioids to patients in both inpatient and outpatient settings.

In general, drugs of abuse act on the mesolimbic dopaminergic pathway, originating in the ventral tegmental area (VTA) of the midbrain and projecting to the nucleus accumbens (NAc) in the ventral striatum, where activation of the mesolimbic pathway is accompanied by the perception of reward (Adinoff, 2004). Furthermore, a three-stage cycle of SUD has been hypothesized which consists of the binge/intoxication stage, the withdrawal/negative affect stage, and the preoccupation/anticipation stage (Koob and Volkow, 2016). Each of these stages results in neuroplastic changes in the brain reward system. The binge/intoxication stage is mediated by the basal ganglia and represents dysfunction with incentive salience or pathological habits. The withdrawal/negative affect stage represents the negative emotional states associated with opioid addiction and is mediated by the extended amygdala. Lastly, the preoccupation/anticipation stage represents dysfunction in executive function, as executive control over incentive salience is essential to break the addiction cycle. The preoccupation/anticipation stage is mediated by the prefrontal cortex (PFC) (Koob and Volkow, 2016). OUD follows this three-stage cycle and individuals who develop OUD have a chronic remitting course of the disorder, where they can cycle in and out of active OUD over years or decades. In addition to the acquisition being broadly based on reward, the withdrawal/negative affective states keep users in the cycle of addiction. The withdrawal/negative affective states particularly consist of key motivational elements including chronic irritability, emotional pain, dysphoria, states of stress, and loss of motivation for natural rewards (Koob and Volkow, 2016). Therefore, with chronic opioid use, anti-reward circuits can be activated and involve overactivation of the habenula or overactivation of the dynorphin system in the ventral striatum, which can decrease dopamine neuron firing (Koob and Volkow, 2016). Furthermore, as tolerance and withdrawal develop, the brain’s stress systems such as the hypothalamic-pituitary adrenal (HPA) axis can become activated and release factors such as corticotropin-releasing factor (CRF), norepinephrine, and dynorphin. The stress-derived factors are then recruited in the extended amygdala and contribute to the development of the associated negative emotional states (Koob and Volkow, 2016). Additionally, in the context of withdrawal when drug intake ceases, direct CRF release in the extended amygdala increases and parallels the somatic and psychological withdrawal signs (Logrip et al., 2011). Consequently, the combination of an initial increase in reward, followed by a decrease in reward function as well as an increase in stress function, significantly contributes to the compulsive drug seeking behavior and addiction.

Due to the impact of the opioid epidemic nationwide, it is imperative to gain insight into the underlying mechanisms behind drugs of abuse, especially opioid use. A potential mechanism underlying SUD encompasses elevated inflammatory processes mediated by the activation of the microglial cells. Various investigations have implicated the microglia, the brain parenchymal macrophages, in drug-induced neuroinflammation and have investigated the role of the microglia in addiction-related neuronal circuits (Kovács, 2012). Microglial cells possess several receptors, which allow them to respond to several different stimuli such as local signals in the central nervous system (CNS) as well as distant signals from the gastrointestinal tract. In particular, the gut microbiome, the community of bacteria residing in the gut, was found to modulate microglial immune programming (Erny et al., 2015). Therefore, how the gut microbiome communicates with the microglia and results in their activation during drug abuse may be an important modulator of addiction-related circuitries and behaviors. In this review, we will explore the role of microglia and their connection to the gut microbiome in the context of SUD with various drugs of abuse, with a particular focus on opioids.

## 2 Microglia

### 2.1 Microglial function and development

Microglia are the innate immune cells of the CNS that function as macrophages with great morphologic and functional plasticity. These glial cells comprise approximately 10–15% of all glial cells in the CNS (Nayak et al., 2014). Unlike other cells of the CNS, microglia are established almost entirely from yolk-sac erythromyeloid progenitors that reside in the surroundings of the neuroepithelium during development, well before other glial cells (Ginhoux et al., 2010). These erythromyeloid progenitors then develop into microglia progenitors and invade the epithelium via the circulatory system using matrix metalloproteinases. During this stage of development, microglia have a more free-form amoeboid morphology, which eventually converts into the classic ramified morphology by postnatal day 28 (Nayak et al., 2014). Adult microglia are characterized by their unique motility and structural responsiveness to stimuli (Davalos et al., 2005). Upon maturation, microglia play important roles as immune cells as they detect pathogens and promote repair in line with traditional macrophage function (Butovsky et al., 2006). Additionally, microglia play other essential roles in several routine neural processes, including neurogenesis, synaptic pruning, and neuroinflammation (Cowan and Petri, 2018). Resting microglia can act as support cells by interacting with surrounding neurons and releasing soluble factors that are involved in maintaining synaptic plasticity. The non-immune supervisory role of microglia is important in long-term potentiation, learning, and memory (Parkhurst et al., 2013).

### 2.2 Microglial activation

During normal physiological function, microglia remain in a resting state, where they mediate the health of local tissue by using their processes to monitor neurons and synapses (Helmut et al., 2011). However, upon disease or injury, microglia undergo distinct changes in morphology upon binding of a stimulus in a process known as microglial activation. Microglial activation refers to a change of conformation into an amoeboid shape with a reduction of cellular processes as well as an increased production of factors such as cytokines, reactive oxygen species (ROS), and protein markers in these abnormal physiological states. Structurally, microglia express toll-like receptor (TLR) 4, which recognizes pathogen-associated molecular patterns (PAMPs). PAMPs can include cell surface components of microbes such as lipoproteins and nucleic acids. Common PAMPs investigated in the study of TLR4 activation and inflammatory processes are lipopolysaccharides (LPS), often found on the outer membrane of Gram-negative bacteria. Microglial TLR4 associates with myeloid differentiation factor 2 (MD2), forming a complex onto which LPS binds, causing microglia to adopt an activated state. Upon LPS binding, intracellular mitogen-activated protein kinase (MAPK) activity increases via a MyD88-dependent cascade (Akira et al., 2006; Wang et al., 2012; Cahill et al., 2016). These MAPKs induce altered protein expression leading to increased production of pro-inflammatory factors such as interleukin (IL)-1β, tumor necrosis factor (TNF)-α, IL-6, IL-12, brain derived neurotropic factor (BDNF), and increased expression of cell surface markers, as well as intracellular inducible nitric oxide synthase (iNOS) (Hu et al., 2012). The overexpression of these factors subsequently affects neurotransmission and plasticity.

## 3 Microglial – neural communication in a drug context

It is of interest to explore the nature of microglial-neural crosstalk, through the action of microglial-released pro-inflammatory factors, to elucidate how microglial activation caused by drug use may be involved in the modulation of neurotransmission to influence the onset of SUD.

### 3.1 Microglial-neural crosstalk in the onset of SUD

Neurons express a range of receptors mediated by microglial-released factors, which is how microglia may influence neurotransmission. This communication between microglia and neurons is critical for the maintenance of normal physiological brain functions during development and adulthood. For example, the microglial-released factor, TNFα, has been associated with increased neuronal AMPA receptors in the hippocampus, influencing synaptic plasticity and memory (Stellwagen and Malenka, 2006). In disease or disease-related conditions, such as the chronic use of opioids, the excessive concentration of inflammatory factors such as cytokines TNFα and IL-1B released by activated microglia impair synaptic plasticity in neurons (Lynch, 2015). The released factors can potentially alter GABAergic transmission via stripping of inhibitory synapses (Chen et al., 2014), and modulate glycinergic (Cantaut-Belarif et al., 2017) and glutamatergic transmission (Antonucci et al., 2012). In the present review, we will explore how signaling from the gut microbiome to the CNS may interplay with the effects of the microglial released factors on neural transmission in the context of drug use. It is imperative to explore these effects in the use of various drugs, especially opioids, to further gain insight on the role of microglia in SUD.

### 3.2 TNFα as a pro-inflammatory factor

**FIGURE 1:**
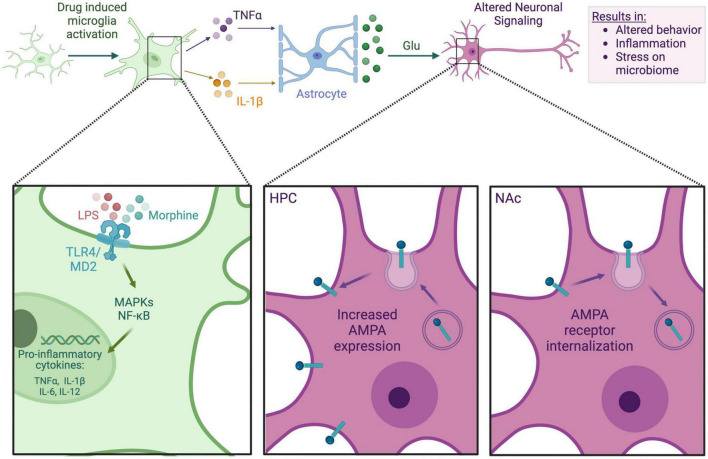
Microglia-neuron interaction in an opioid context. This figure depicts the general mechanism by which morphine may impact neuronal signaling via the microglia. Either LPS from a dysbiotic microbiome or morphine itself binds to microglial TLR4, prompting the release of proinflammatory cytokines such as TNFα and IL-1β. These cytokines bind to local astrocytes, causing glutamate release. Glutamate binds to neuronal AMPA receptors, which are differentially expressed in the hippocampus (HPC) and NAc, causing altered neuronal signaling. Alterations in neuronal signaling can lead to addictive behaviors, neuroinflammation, systemic inflammation, and further stress on the gut microbiome.

As previously mentioned, TNFα is one microglial-released factor that has been shown to mediate the expression of synaptic AMPA receptors, overall synaptic strength, and plasticity (Lewitus et al., 2016). The literature is mixed in terms of the role of TNFα in AMPA receptor expression. While some studies find that TNFα upregulates AMPA receptor expression, others find that TNFα decreases AMPA receptor expression. However, studies have shown that TNFα-mediated AMPA receptor upregulation primarily occurs in the hippocampus (Leonoudakis et al., 2008; He et al., 2012), while TNFα-mediated AMPA receptor downregulation occurs in the NAc (Lewitus et al., 2016), as shown in Figure 1. Therefore, there may be differential effects of microglial-released TNFα in various regions of the brain. In general, drugs of abuse can lead to the TLR4/MD2 complex of microglia. Cocaine, for example, has been shown to elicit TNFα release by activation of microglia in mice, driving the internalization of synaptic AMPA receptors in the NAc, decreasing synaptic strength, and causing the subsequent suppression of behavioral sensitization, as measured by drug-induced locomotor response, to repeated administration of cocaine (Lewitus et al., 2016). Walter and Crews (2017) demonstrated that binge ethanol withdrawal also increases brain TNFα in mice. This effect was found to be dose dependent, in which higher doses of binge ethanol led to higher microglial pro-inflammatory cytokine expression during withdrawal (Walter and Crews, 2017). Opioids have also been shown to elicit TNFα release through microglial activation in mice (Merighi et al., 2013). Using a TNFα antagonist has interestingly been shown to suppress morphine tolerance in rats, highlighting a potential connection between the released pro-inflammatory factors such as TNFα and the cycle of addiction (Shen et al., 2011). TNFα released by activated microglia can also have effects on local glia, such as causing the release of glutamate from local astrocytes (Zhang et al., 2020). The changes in receptor expression and increased cytokine release in various drug contexts could be a potential explanation of how released inflammatory factors from activated microglia shape neurotransmission to impact various stages in the cycle of addiction, such as withdrawal and tolerance.

### 3.3 IL-1β as a pro-inflammatory factor

There is also research that suggests the role of IL-1β in microglial drug activation. Bilbo et al. (2005) demonstrated in neonatal rats that a postnatal dose of *Escherichia coli* caused increased microglial activation and excessive IL-1β release in the hippocampus and cortex. Rats that were treated neonatally with *E. coli* were treated in adulthood with LPS, which resulted in more rapid overproduction of IL-1β and impaired learning and memory functions (Bilbo et al., 2005). This impairment was not seen when adult rats were treated with caspase I inhibitor, which blocked the production of IL-1β (Bilbo et al., 2005). While IL-1β at lower physiological concentrations is required for learning and memory, excessive concentrations impair learning and memory functions in the hippocampus and cortex, which may play a role in the addiction process. Overall, data imply that a homeostatic concentration of IL-1β is required to maintain normal synaptic plasticity, learning, and memory.

Another study focused on nicotine withdrawal in mice found that chronic nicotine treated mice as well as those undergoing withdrawal showed altered microglial morphology in the NAc (Adeluyi et al., 2019). Moreover, nicotine withdrawal in mice showed elevated TNFα and IL-1β mRNA levels and ROS production in the NAc compared to saline and chronic nicotine treatments, suggesting an association between microglia-released factors and the withdrawal state specifically. However, these results were not observed in the caudate putamen, which showed a significant decrease in IL-1β mRNA expression during withdrawal, but no significant change in TNFα mRNA expression between treatment groups. Results from this study indicate differential microglial inflammatory responses from chronic nicotine treatment and withdrawal treatment as well as a brain region dependent response. The study also found that chronic nicotine withdrawal was associated with an anxiogenic effect in mice using a marble-burying and open field test (Adeluyi et al., 2019). This effect was not seen in mice undergoing withdrawal that were treated with PLX5622, a colony-stimulating factor–1 receptor (CSF1R) inhibitor, which pharmacologically depletes microglia (Adeluyi et al., 2019). Anxiety is a symptom of withdrawal associated with the function of the NAc, further supporting the idea that microglial function within the NAc in involved in drug withdrawal. While this study focused on the action of nicotine, additional studies found similar effects of microglia activation after morphine treatment (Merighi et al., 2013; Schwarz and Bilbo, 2013). For example, increased levels of pro-inflammatory factors IL-1β and TNFα are also expressed by microglia in the NAc after chronic morphine treatment. Therefore, hypotheses could be drawn that the nicotine withdrawal processes via microglial action in the NAc, a major center for addictive processes, could be similar to those for opioid dependence and withdrawal. Activation of opioid receptors in this region would theoretically elicit a similar microglial release of cytokines, such as IL-1β and TNF-α, influencing neuronal transmission and potentially mediating opioid reward and withdrawal. In fact, chronic morphine exposure has been reported to be associated with elevated plasma levels of IL-1β (Ocasio et al., 2004). Since microglia play key roles in maintaining synaptic function and overall plasticity, microglial activation and released cytokines may be important mediators in the addiction cycle.

### 3.4 The role of microglia in characteristic opioid use disorder behavior

While research has shown the involvement of microglia with the use of several drugs, opioids are of particular importance due to the ongoing opioid epidemic, which has deleteriously affected a large population nationally (Centers for Disease Control, [CDC], 2021). The involvement of microglia in opioid use, through activation and subsequent neurotransmission modulation, may potentially play a significant role in the behavioral characteristic of OUD, such as tolerance. Opioid tolerance specifically occurs when repeated use of opioids results in higher dosages required to elicit the same level of pain relief as before. Activated microglia have been shown to play a role in morphine tolerance through the release of pro-inflammatory cytokines including IL-1β and TNF-α. IL-1β is of particular importance as it has been shown to be primarily involved in the onset of morphine tolerance, and inhibition of IL-1β signaling has been shown to prevent tolerance in mice (Shavit and Wolf, 2005). TNFα antagonists have been shown to restore the analgesic effects of morphine in rats (Kawai and Akira, 2007), in line with other studies showing TNF-α playing a role in mediating a mu-opioid receptor- PKCε (MOR-PKCε) signaling pathway in mouse activated microglia in morphine tolerance (Merighi et al., 2013). Another study treated adolescent rats with either saline or morphine to investigate the effect of morphine on the drug-induced reinstatement of conditioned place preference (CPP) later in adulthood (Schwarz and Bilbo, 2013). Morphine re-exposure in adulthood in the group that had received morphine in adolescence elicited strong reinstatement of CPP. This effect was not seen in rats treated with ibudilast, an inhibitor of morphine-induced cytokine and chemokine production from microglia (Schwarz and Bilbo, 2013). These data generally indicate that morphine exposure during adolescence increased TLR4 expression of microglia in the NAc, a brain region implicated in drug reward. Increased baseline TLR4 production results in the increased production of pro-inflammatory factors from microglia upon morphine binding. The inhibitory effect of ibudilast on the reinstatement of morphine CPP supports the conclusion that microglia are involved in the long-term increase in the risk of reinstated drug-seeking behavior after a primary exposure (Schwarz and Bilbo, 2013). Other studies show that long-term morphine administration causes general proliferation of glia (Hutchinson et al., 2009; Schwarz et al., 2013). While the exact mechanisms underlying the relationship between microglial activation and its involvement in opioid-induced reward and reinstatement are not fully researched, the relationship between the release of pro-inflammatory factors from activated microglia in the NAc and the effect on neurotransmission might elucidate specific pathways for further research in the circuitry behind drug-seeking behavior and the SUD. Microglia also play other roles in the CNS; they act as support cells that aid in long term potentiation, learning, and memory. While the interaction between immune-role microglia and alternate-role microglia is not well-researched, it could be hypothesized that drug-seeking behavior and SUD may also be impacted by their interaction (Parkhurst et al., 2013). Future research on the interaction between microglia with various roles in the modulation of neurotransmission in OUD could fill in this missing link. Taken in conjunction, the results of various studies investigating the activation of microglia in a drug context show a general effect of released microglial pro-inflammatory factors on neuromodulation, with the NAc as a major area of interest. Based on these studies, it could be hypothesized that the NAc, a key structure in the mesolimbic reward pathway, plays an important role in drug-seeking behaviors and in the onset of SUD through activated microglia-neural crosstalk, as summarized in Figure 1.

## 4 Gut microbiome and microglia

### 4.1 Importance of the gut microbiome

In addition to the microglia-neural crosstalk, recent research has uncovered a potential connection between the gut microbiome and microglial functioning. Over the years, the gut microbiome has revealed itself to be a key modulator of health and disease (Kinross et al., 2011). The gut microbiome encompasses trillions of microorganisms of different species and genes (Tanaka and Nakayama, 2017). The microbes in the gut include bacteria, fungi, parasites, and viruses that can be both helpful and harmful (Tanaka and Nakayama, 2017). Symbiotic and commensal bacteria are beneficial, as they support the homeostatic health of the body; whereas, pathogenic bacteria can promote disease and impair homeostatic functioning (Kinross et al., 2011). A healthy gut microbiome, where pathogenic bacteria and symbiotic microbiota coexist normally, functions to metabolize polysaccharides and toxic products such as heavy metals through absorption, metabolism, sequestration, and excretion (Liu et al., 2020). The microbiome also serves as a barrier against pathogens by promoting epithelial cell maturation and angiogenesis of the intestinal barrier (Human Microbiome Project Consortium., 2013). The gut microbiome also significantly contributes to the post-natal development of the immune system. Studies performed on germ free (GF) mice, which were raised in the absence of live microbes, revealed the critical role of the microbiome on the development of the immune system. GF mice were found to have a reduced number of CD4^+^ T cells and IgA producing plasma cells, which indicated the microbiome’s involvement in secondary and lymphoid structure development (Bauer et al., 1963; Mazmanian et al., 2005). Additionally, the microbiota plays a substantial role on the immune cells of the CNS, the microglia, as prenatal and postnatal inputs from the gut microbiota are critical for microglial maturation and function (Abdel-Haq et al., 2019). GF mice were found to display global defects in microglia with altered cell proportions and an immature phenotype which lead to impaired innate immune responses (Erny et al., 2015). The gut microbiome’s role in the development of the immune system has important and long-term implications for human health. Various studies have revealed that alterations of the microbiota of either mothers or neonates may predispose the neonates to dysregulated immune responses and related diseases such as asthma and allergies (Böttcher et al., 2000; Ege et al., 2011). Overall, the symbiotic relationship between commensal microbiota and the host achieves a balanced and mutually beneficial state necessary for the effective development and function of several systems, particularly of the microglia in the CNS.

### 4.2 Routes of communication between the gut microbiome and the brain

**FIGURE 2:**
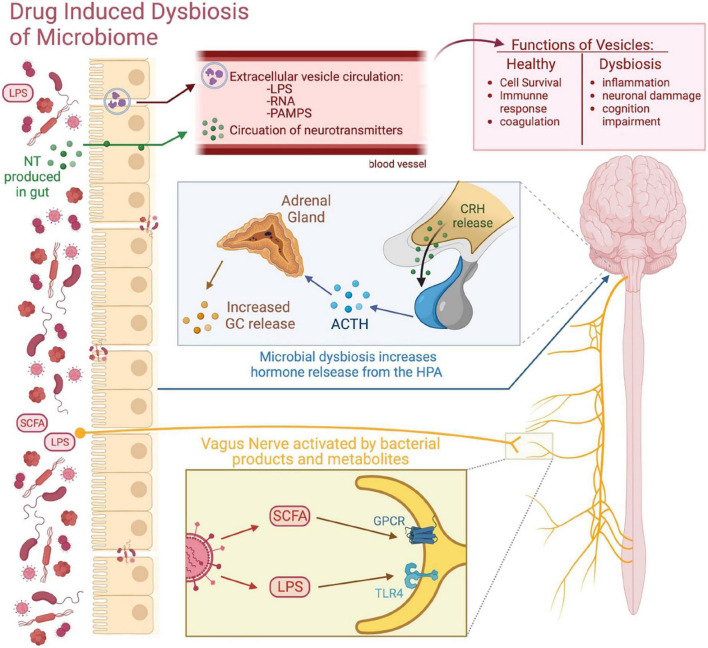
Routes of communication between the gut microbiome and the brain. This figure visually depicts the different routes of communication between the gut microbiome and the brain. Microbial communication with secreted neurotransmitters (NT) and microbiome-derived EVs allow for the transfer of bacterial products into the CNS. The gut microbiome can influence brain activity through the HPA axis by influencing the secretion of its various hormones. The vagus nerve acts as a neuroanatomical connection between the gut and the CNS, as gut bacterial products and metabolites such as LPS and SCFAs can bind to their receptive receptors expressed on the vagus nerve and send signals to brain.

There are many theories on how the gut microbiome communicates with the brain which involve the vagus nerve, the HPA axis, and extracellular vehicles (EVs) (Figure 2). The vagus nerve is one of the known communication mechanisms which acts as a neuroanatomical pathway between the gut microbiome and the brain (Forsythe et al., 2014). The vagus nerve, the longest of the 12 cranial nerves, extends into all the layers of the digestive wall but does not cross the epithelial layer (Wang and Powley, 2007). Therefore, the vagal nerve fibers are not in direct contact with the gut luminal microbiota but instead indirectly receive signals from the microbiota as bacterial products and metabolites which diffuse across the epithelial layer. Short-chain fatty acids (SCFAs) and LPS produced by the bacterial communities within the lumen indirectly activate the vagal nerve by binding to their receptive receptors, G protein-coupled receptors (GPCR) and TLR4, respectively, expressed on the vagal nerve fibers (Goehler et al., 1999; Bonaz et al., 2018). Additionally, other cells in the epithelium, such as enteroendocrine cells (EECs), which consist of 1% of the intestinal epithelial cells, can help to relay luminal signals to the vagal nerve (Bonaz et al., 2018). The EECs also express receptors for the SCFAs and TLRs for the recognition of bacterial products. The EECs then release serotonin and activate 5-HT3 receptors on vagal afferent fibers, allowing the vagus nerve to effectively sense gut bacterial products and metabolites and then send signals back to the brain (Li et al., 2000).

Another potential route of communication between the gut microbiota and the brain is through the HPA axis of the endocrine system. The HPA axis is a robust neuroendocrine system that mediates the effects of stressors by regulating numerous physiological processes, such as metabolism, immune responses, and the autonomic nervous system (Sheng et al., 2021). The HPA axis begins with the activation of the hypothalamus, particularly the paraventricular nucleus, which releases CRF and induces the anterior pituitary gland to release adrenocorticotropic hormone (ACTH). ACTH then stimulates the adrenal cortex to produce Glucocorticoids (GCs) (Sheng et al., 2021). GCs such as cortisol bind to GC receptors present on many cells throughout the body, including microglial cells, to result in various homeostatic functions. The GC signaling system has been found to play a critical role in the development of addiction. Intravenous self-administration of cocaine and reinstatement was found to depend on GC levels (Deroche and Marinelli, 1997), indicating the GC receptor as a potential therapeutic target in the treatment or prevention of cocaine abuse (Deroche-Gamonet et al., 2003). Daily changes in the level of GCs maintain normal homeostasis by increasing vascular tone, alertness, energy and priming the immune system to defend the body (Sheng et al., 2021). Stressful events or injury elevate GC levels and activate the “fight-or-flight” defense response. The adrenal medulla, part of autonomic nervous system (ANS), responds to stressors and releases epinephrine and norepinephrine into the bloodstream, which increases heart rate, blood pressure, and metabolism (Sheng et al., 2021). Furthermore, the HPA axis is equipped with a negative feedback system which consists of circulating levels GCs feeding back to the hypothalamus and the pituitary gland and blocking secretion of CRF and ACTH, respectively, in order to terminate the stress response (Sheng et al., 2021). Such regulated hemostatic responses are important to maintain the normal functioning of the body. In fact, HPA axis dysregulation results in various downstream physiological consequences including dysregulated endocrine, neural, and behavioral responses to acute stress (Kinlein et al., 2015). Consequently, the association between the gut microbiota and the HPA axis have been an active area of investigation. Evidence shows that the gut microbiome develops in parallel to the HPA axis (de Weerth, 2017) and that HPA axis activation impacts the gut microbiota. In animal models, various stressors including maternal separation, social stressors, and hostile cage environment have been found to impact the gut microbial composition and lead to microbial alterations such as the decrease in commensal bacteria *Lactobacilli* and *Bacteroides* (Tannock and Savage, 1974; Bailey et al., 2011; Cussotto et al., 2018).

Studies performed on GF mice, lacking detectable microbiota, demonstrate that the gut microbiome influences the HPA axis and the stress response. GF mice exposed to acute restraint stress exhibited an exaggerated response of the HPA axis with elevated plasma ACTH and corticosterone levels (Sudo et al., 2004). Interestingly, the exaggerated HPA stress response by GF mice was reversed by reconstitution with *Bifidobacterium infantis* and specific pathogen-free (SPF) feces at an early stage, but not by any reconstitution exerted at a later stage (Sudo et al., 2004). These results suggest that commensal microbiota can have lasting effects on the postnatal development of HPA stress response in mice. Similar exaggerated responses of the HPA axis were observed in response to acute novel-environment stress in both GF mice (Clarke et al., 2013) and GF rats (Crumeyrolle-Arias et al., 2014). Vagnerová et al. (2019) investigated the effect of the microbiota on the distinct structures of the HPA axis such as the pituitary, adrenal gland, and intestine. The authors show that in addition to the plasma corticosterone response to acute restraint stress being higher in GF than in SPF mice, the interaction between stress and the microbiota during activation of glucocorticoid steroidogenesis differed between the HPA axis organs. In particular, a lower expression of pro-opiomelanocortin (*Pomc)* and the gene encoding CRF receptor type 1 *(CRFr1)* in the pituitary of SPF mice was found to partially explain the exaggerated HPA axis reactivity in GF animals (Vagnerová et al., 2019). Additionally, pregnant women with a history of childhood adversities were found to have greater differential abundance of the pathogenic bacteria *Prevotella* and the abundance of several bacterial taxa was associated with higher cortisol levels (Hantsoo et al., 2019). More importantly, probiotic interventions were shown to have beneficial effects on the HPA axis. Administration of *Lactobacillus helveticus* R0052 and *Bifidobacterium longum* R0175 significantly reduced anxiety-like behavior in rats and alleviated psychological distress in human volunteers, which was accompanied by a decrease in cortisol levels (Messaoudi et al., 2011). In addition, the administration of the *Bifidobacterium longum* 1714 strain not only reduced the level of stress but also improved memory in healthy volunteers (Allen Hutch et al., 2016). Therefore, the gut microbiome influences the HPA axis and significantly participates in the developmental programming of stress responses (Walker and Spencer, 2018). Importantly, exposure to either acute or chronic stress has been found to induce robust microglial activation in the brain (Sugama and Kakinuma, 2020). Stress-induced microglial activation was found to occur through β-adrenergic receptor action with noradrenaline as a key neurotransmitter (Sugama et al., 2019). These data show that the gut microbiome can influence brain activity and microglial activity through the HPA axis by influencing the endocrine system and the secretion of its various hormones.

More recently, a route of communication between the gut and brain has been found to involve EVs derived from bacteria (Cuesta et al., 2021). Several organisms, including bacteria, can secrete several types of membrane vesicles or EVs, which are part of the secretome system and are important for intra- and extracellular communication. Normally, EVs contain proteins such as tetraspanins, receptors ligands or adhesion molecules, RNAs, and lipids (Cuesta et al., 2021). Under healthy physiological conditions, EVs are involved in cell survival, immune response, and coagulation (Kalluri and LeBleu, 2020; Veziroglu and Mias, 2020). Bacteria of the gut microbiome can communicate with the brain by secreting neurotransmitters, such as serotonin, dopamine, noradrenaline, and GABA, hormone-like metabolites, and SCFAs that can be transported through their EVs or diffuse across the epithelial barrier (Haas-neill and Forsythe, 2020). Evidence shows that gut bacteria-derived EVs are able to deliver their cargo directly to the CNS (Haas-neill and Forsythe, 2020), which may significantly mediate gut-brain communication and may potentially be utilized in therapeutic interventions (Pirolli et al., 2021). During gut microbial dysbiosis, pathogenic bacterial species increase in abundance and secrete EVs containing molecules that can affect host gene expression such as bacterial small RNAs, LPS, proteins, and other PAMPs which can stimulate host immune and non-immune cells via TLRs to produce systemic innate and adaptive immune responses (Ellis et al., 2010). Additionally, in the context of dysbiosis, the intestinal epithelium is damaged by both bacterial activity and the inflammatory immune response, which further increases epithelial permeability and the transfer of EV components from the intestinal lumen to the bloodstream. Furthermore, a recent report showed that bacterial EVs could cross the blood–brain barrier (BBB) through the vagal nerve (Lee et al., 2020). EVs of the bacteria *Paenalcaligenes hominis* are associated with the onset of cognitive function-impaired disorders, such as Alzheimer’s disease, via penetration to the brain through the blood as well as the vagus nerve (Lee et al., 2020). In addition, there is some evidence that bacterial EVs can themselves increase BBB permeability (Ridder et al., 2014; Muraca et al., 2015), which can further allow for bacterial products to reach the CNS and affect its functions. Moreover, bacterial EVs have been found to directly alter neurologic function and induce pathology in the brain. Gut microbiome-derived EVs were purified from the feces of Alzheimer’s disease (AD) patients and injected into mice (Wei et al., 2020). The results showed increased BBB permeability, increased tau phosphorylation, increased secretion of inflammatory cytokines, activated microglia, and induced neuroinflammation compared to EVs from healthy patients, which did not induce these changes (Wei et al., 2020). Importantly, the development of cognitive dysfunction including learning and memory deficits were also observed in the injected mice (Wei et al., 2020). Another study showed that intracardiac injection in mice of the EVs derived from the main bacteria implicated in the pathology of periodontal disease, *Aggregatibacter actinomycetemcomitans (Aa)*, showed successful delivery to the brain after crossing the BBB and their extracellular RNA cargo was associated with increased expression of TNFα in the mouse brain (Han et al., 2019). Therefore, accumulating evidence show that bacterial EVs can potentially alter behavior and gene expression in the brain. Overall, bacterial EVs from the gut can reach the CNS, where they may activate immune cells, such as the microglia, trigger the release of pro-inflammatory cytokines and cause neuronal damage.

### 4.3 Microbiome-mediated modulation of microglia during drug use

The communication between the microglia and the gut microbiome may be an important factor to consider as a mechanism of microglial activation during drug abuse. Microglial cells are dependent on a healthy and complex microbiome for proper development, maturation, and function. GF mice were found to display global defects in microglia with altered cell proportions and an immature phenotype, leading to impaired innate immune responses (Erny et al., 2015). Eradication of the host microbiota resulted in defective microglia while the recolonization of a complex microbiota partially rescued the defective features of the microglia (Erny et al., 2015). Microglia undergo different stages during development; the effects of a lack of microbiome in the GF mice have been very apparent during these different stages and have been found to be sex specific. The absence of microbiome in GF mice had an impact both prenatally and postnatally and, in a sex-specific manner: prenatally, the microglia of male embryos were found to be more profoundly perturbed whereas in adulthood, the microglia of female adults were found to be more profoundly perturbed (Thion et al., 2018). Microglial density varies significantly in different regions of the brain between sexes, which may contribute to the sex specific differences observed. For instance, in early developmental stages, microglia density in the hippocampus is higher in female brains, whereas it is higher in the amygdala in male brains (Yanguas-Casás, 2020). In addition to the gut microbiome’s critical role during development, there is constant crosstalk between the microbiome and the microglia throughout adulthood. Antibiotic treatment during adulthood, which depletes the gut microbiome acutely, resulted in disruptions in the microglia (Guida et al., 2018; Thion et al., 2018). Particularly, antibiotic treatment disrupted the microglia cells in a sex-specific manner where microglia of the female antibiotic-treated adults were more profoundly perturbed. The female microglial cells appeared to be in a more immune-activated state than in the male antibiotic-treated adults (Thion et al., 2018). Overall, these experiments using the GF mice revealed the long-term effects of microbial depletion on the microglia and the use of antibiotics emphasized the acute effects of microbial depletion on microglial responses. Additionally, the depletion of the microbiome was found to produce widespread region- and state-specific changes in the activation of the neuronal ensembles involved in OUD during both the intoxication and withdrawal states (Simpson et al., 2020). Data showed that antibiotic treatment produced robust alterations in the sensitivity of multiple brain regions including the basolateral amygdala (BLA), the periaqueductal gray (PAG), the locus coeruleus (LC), and the central nucleus of the amygdala (CeA), which are involved in oxycodone intoxication and withdrawal (Simpson et al., 2020). Furthermore, probiotic intervention was found to reduce the antibiotic-induced microglial activation (Guida et al., 2018). Consequently, the microbiome may play a key role in the modulation of the brain response to drugs.

Furthermore, drug intake has been found to induce disruptions in the gut microbiome and result in microbial dysbiosis. Opioid-induced microbial changes include the loss of several beneficial microbes such as *Bacteroidales* and the expansion of the pathogenic ones such as *Enterococcus, Sutterella*, and *Clostridium* (Wang et al., 2018). Other changes include disruptions in the gut itself including the epithelial layer and the tight junction proteins which make up the protective barrier between the microbes and the rest of the body (Meng et al., 2013). In particular, morphine treatment was shown to upregulate TLR expression in epithelial cells of small intestine, inducing disruption of tight junctions between epithelial cells and increasing gut permeability (Meng et al., 2013). Tight junction proteins have been shown to seal the gap between gut epithelial cells and play an important role in preventing potential pathogen invasion (Schulzke et al., 2009). Consequently, the morphine-induced disruptions in the epithelial layer of the gut resulted in an increased bacterial translocation and inflammation in the small intestine (Meng et al., 2013). Translocated bacteria in gut epithelium can cause abnormal pro-inflammatory cytokine production including TNFα, IL-1B, and IL-6, another pro-inflammatory cytokine which can further cause disruption of tight junction proteins in gut epithelium (Bruewer et al., 2003). IL-6 is produced by enteric neurons in a regulatory manner to mediate the number and phenotype of microbe-responsive regulatory T (Treg) cells in the gut (Yan et al., 2021). Therefore, opioid exposure and opioid induced microbial dysbiosis is associated with abnormal IL-6 production (Bertolucci et al., 1997; Houghtling and Bayer, 2002), which may alter the equilibrium between the enteric neurons, Treg cells, and gut microbes to modulate the normal host-microbe interface. Additionally, our laboratory recently showed an acute increase in IL-6 after morphine treatment caused a delay in gastric emptying and an accumulation of acid, which resulted in gastric inflammation (Ghosh et al., 2022). Morphine-treated IL-6 KO mice did not exhibit gastric inflammation (Ghosh et al., 2022) but did exhibit reduced opioid responses (Bianchi et al., 1999). Overall, opioid-induced microbial dysbiosis can result in inflammation in the gut, systemic inflammation throughout the body, and even sepsis (Hilburger et al., 1997). Interestingly, IL-6 and IL-10 can help distinguish between Gram-negative and Gram-positive bacterial infections. When compared with healthy people, IL-6 and IL-10 were significantly increased in Gram-negative bacterial infections, while only IL-6 was significantly increased Gram-positive bacterial infections (Zhu et al., 2022). Furthermore, many pro-inflammatory cytokines including IL-6, IL-1α, IL-1β, and TNFα have saturable transport systems to cross from the blood to the CNS (Banks et al., 1995). Thus, several of the peripheral cytokines released in response to an inflammatory event in the gut can directly cross the BBB, reach the brain, and potentially result in neuroinflammation.

Several studies outside the context of drug use demonstrate that pro-inflammatory cytokines released from a dysbiotic gut are associated with neuroinflammation. An altered gut microbiome in a mouse model of Gulf War (GW) Illness was shown to be associated with neuroinflammation via leaky gut and TLR4 activation (Alhasson et al., 2017). Alhasson et al. (2017) showed that IL-1β and monocyte chemoattractant protein-1 (MCP-1) were significantly elevated in the frontal cortex and small intestine of the mice treated with GW chemical. Results also showed tyrosine nitration in the brain and intestinal tissues, a marker for both nitrative and oxidative stress which are integral causes of neuronal and intestinal inflammation (Alhasson et al., 2017). Additionally, gut dysbiosis induced by prolonged antibiotic treatment was shown to activate and proliferate the microglia and impair synaptic transmission in the in the hippocampal CA3 and CA1 subregions (Çalışkan et al., 2022). Since a dysbiotic microbiome results in neuroinflammation and opioid use causes similar dysbiotic disruptions, it is possible that opioid-induced alterations in the gut microbiome may similarly lead to neuroinflammation.

There is a lack of definitive research on the exact mechanisms by which neuroinflammation is mediated by the microbiome. However, Vargas-Caraveo et al. (2017) demonstrated that LPS derived from the lung microbiome can cross the BBB via lipoprotein receptors. The LPS molecules were shown to bind to TLR4 on microglia and astrocytes inducing a proinflammatory response in the brain. Therefore, gut microbiome-derived molecules may also cross the BBB, activate the microglia, and result in neuroinflammation mediated by the release of the pro-inflammatory cytokines. This neuroinflammation may contribute to the behavioral consequences of drug use. Major depression, a co-morbidity of drug abuse, has been found to correlate significantly and positively with pro-inflammatory cytokines production and various indices of systemic immune activation (Maes, 1993). Gut dysbiosis inducing neuroinflammation in the brain may be a significant contributing factor in the development of symptoms associated with chronic opioid use. A study exploring how opioid treatment and cessation impacts the gut microbiome found the gut microbiota to mediate reward and sensory responses associated with morphine dependence (Lee et al., 2018). Lee et al. (2018) showed that intermittent morphine treatment resulted in microglial activation in the VTA and dorsal spinal cord as well as hyperalgesia and impaired reward response. Additionally, depletion of the gut microbiota via antibiotic treatment similarly altered microglial morphology, and led to hyperalgesia and impaired reward behavior (Lee et al., 2018). Interestingly, gut microbiome recolonization from naïve mice donors, but not morphine-dependent donors, restored normal reward behavior and microglia morphology in the antibiotic-treated mice (Lee et al., 2018). Overall, alterations in the gut microbiome may activate microglia to release pro-inflammatory cytokines inducing neuroinflammation, which can interact with excitatory transmission and remodel synapses involved in drug-related behaviors. Therefore, further investigations on the intersection between the gut microbiome, the microglia, and OUD would elucidate the exact mechanisms by which alterations in the gut microbiome can drive neuroinflammation and modulate the behavioral consequences of drug use.

## 5 Summary

Substance use disorder, under which OUD is categorized, is a disease that affects millions of individuals with minimal therapeutic treatment options. The lack of treatment is in part due to the complexity of the disease and the lack of full understanding of the mechanisms of long-term drug use. Hopefully, research investigating the mechanisms of drug use will provide new therapeutic options to those struggling with SUDs. A promising area of research is the interplay between microglial activation and the damaged gut microbiome during drug use. This gut-microglial communication leads to altered brain signaling in drug-related behaviors and can have negative consequences on the entire body.

**FIGURE 3:**
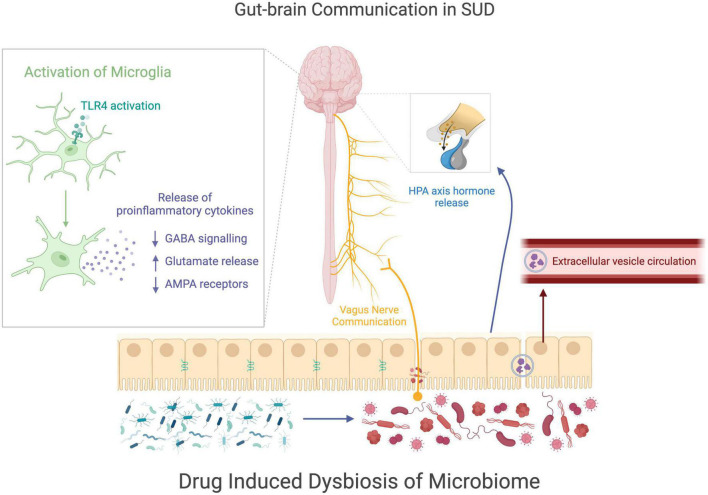
Gut-brain communication in SUDs. This figure visually summarizes how drug induced changes to the microbiome may be related to cellular changes in the brain during drug use. Drug use causes a disruption of gut homeostasis, resulting in a dysbiotic state of the microbiome. The microbial change in the gut may alter the communications sent from the gut to the nervous system through the vagus nerve, the HPA axis, or extracellular vesicles. The communication between the gut and the nervous system may contribute to the increase of microglial activation, which alters astrocyte and neuron signaling, potentially modifying behaviors comorbid with drug use.

Since it is most likely that a combination of mechanisms communicates to produce the complexity of SUDs, this review highlights the communication of the gut microbiome and the microglia to discuss how these systems could be connected in the context of these disorders. There is sufficient research to support that the gut and brain can communicate with one another, and that the microbiome can even have influence on the morphology and activation of microglia. A drug induced dysbiosis of the microbiome may cause altered communication between the gut and brain, thus leading to the development of SUDs (Figure 3). The dysbiosis of the microbiome during drug use causes an increased abundance of pathogenic bacteria, and impaired gut barrier integrity, thus altering the way the gut communicates with the rest of the body. The disruption of the gut homeostatic environment may interfere with the metabolite signaling of the vagus nerve, resulting in alternative metabolites activating the nerve or just a change in amount of activation, either of which could affect the brain downstream. Disruptions to the gut homeostasis could also impact the communication with the HPA axis, resulting in a change of hormone release during drug use. The increase in pathogenic bacteria also results in harmful products being released into the bloodstream via EVs, which can either affect the brain directly through the bloodstream or through additional vagal nerve signaling. The altered routes of communication between the gut and brain may explain the change in microglia activity seen during drug use, as the microbiome has been documented to modulate microglial function. The pathogenic bacteria can result in the indirect signaling of TLR4 on the microglia cells, causing the morphology to change and the cells to become activated.

In the state of activation, after drug use, microglia release pro-inflammatory cytokines which leads to a modulation of neurotransmission through altered GABA signaling, glutamate release, and AMPA receptor expression, with the NAc being a particular area of interest in the reward pathway. The changes in brain signaling could contribute to the behavioral consequences of drug use as well as contribute to the onset of SUDs, however, additional research is needed to fully understand the impact of microglia activation on SUDs. Additionally understanding how the gut microbiome contributes to the changes in the microglia, could result in new therapeutic targets for treating SUD, particularly OUD, by intervening at the level of the gut microbiome.

## 6 Conclusion

The premise of our review is that drug exposure alters both microglial and gut microbiome functioning and communication via the gut-brain-axis and may additively drive addictive behaviors. Microglia play important roles not only in the normal homeostasis of brain function, but are also highly involved in SUD, under which OUD is of particular focus in this review. Accumulating evidence shows that activated microglia release pro-inflammatory factors in various drug abuse models. Consequently, the effects of these microglial pro-inflammatory factors on neuromodulation in the context of drug abuse is a major area of interest. In addition to drug exposure acting directly on microglia, drug intake causes disruptions in the gut microbiome and the gut barrier, causing gut inflammation and bacterial translocation, resulting in both systemic inflammation and neuroinflammation through microglial activation. Thus, activation of the microglia in the brain may represent a response to both the inflammatory event in the gut and the broader systematic inflammatory response. The connection between the gut microbiome and microglia may be mediating the onset and characterization of OUD. Therefore, it is important to further investigate the specific mechanisms behind these proposed opioid-gut-microglial connections as they could be used as potential therapeutic targets. Both microglial- and microbiome-targeted interventions could help to reduce the burden of SUDs, and in particular, OUD amidst the ongoing opioid epidemic.

## Author contributions

BT, GV, DA, and SR: conceptualization and writing—review and editing. DA and GV: literature review and collection and writing—original draft preparation. BT: visualization. SR: funding acquisition. All authors have read and agreed to the published version of the manuscript.
